# Behavioral changes of patients after orthognathic surgery develop on the basis of the loss of vomeronasal organ: a hypothesis

**DOI:** 10.1186/1746-160X-5-5

**Published:** 2009-01-22

**Authors:** René Foltán, Jiří Šedý

**Affiliations:** 1 Department of Stomatology, First Faculty of Medicine and General Teaching Hospital, Charles University, Prague, Czech Republic; 2 Institute of Experimental Medicine and Institute of Physiology, Academy of Science of the Czech Republic, Prague, Czech Republic

## Abstract

We introduce a hypothesis which presumes that damage to the vomeronasal organ during a Le Fort I osteotomy of the maxilla for the purpose of orthognathic surgical treatment of congenital or acquired jaw deformities affects the patient's social life in terms of the selection of mates and establishment of relationships. The vomeronasal organ is chemosensory for pheromones, and thus registers unconscious olfactory information which might subsequently act on the limbic system of an individual and influence the selection of mates. We believe it is connected to an inhibitory feedback mechanism which is responsible for the exclusion of inappropriate mates. When the vomeronasal organ is removed or damaged during a maxillary osteotomy, the inhibitory function is lost, the patient loses the involuntary ability to exclude inappropriate mates, may become less committed to an existing mate, or even become promiscuous.

## Background

Orthognathic surgery is a surgical discipline aimed at correcting congenital or acquired jaw deformities. It may be indicated for functional and/or aesthetic reasons. Clinical situations demanding such correction include the need for reconstruction of biting and chewing functions [1], the correction of sleep apnea syndrome [2-5], temporomandibular joint disorders [6], or cleft palate [7]. Orthognathic surgery is primarily based on osteotomy of the facial bones and advancement or set back of the upper and/or lower jaw bone. Maxillary osteotomy is performed in fracture line Le Fort I, originally described by Rene Le Fort [1,6].

Orthognathic surgery significantly affects the psychological aspects of a patient's personality in almost all cases. In the majority of cases, it significantly increases the patient's self-confidence [8]. Retrospective studies suggest a very high level of satisfaction following orthognathic surgery in comparison with other types of cosmetic surgery, such as rhinoplasty or breast augmentation/reduction [9]. It has been shown that patients significantly improved their psychological-psychiatric profile, including psychoses, neuroses, personality disorders, and social integration [10]. Most importantly for our study, physical attractiveness has a considerable impact on the establishment of new relationships, including dating [11].

In mammals, the vomeronasal organ (VNO), also known as Jacobson's organ, is a chemosensory organ, the function of which is still not precisely known. In some phylogenetically older animals, such as mice or rats, the VNO is most likely used in the detection of pheromones, i.e., a chemical substance which carries a message about the physiological or behavioral state of a living organism to members of its own species, resulting in a specific reaction [12,13]. Connections between the VNO and the amygdala and limbic system have been described, both of which are recognized as the seat of emotional, hormonal, and autonomic control [14]. For example, snakes use this organ to sense prey, sticking their tongue out to gather scents and touching it to the opening of the organ when the tongue is retracted [15]. Elephants transfer chemosensory stimuli to the vomeronasal opening in the roof of their mouths using a prehensile structure, sometimes called a "finger," at the tip of their trunks. Some mammals, such as horses, use a distinctive facial movement, referred to as the flehmen response, to direct inhaled compounds to this organ. House cats often may be seen making a grimace when examining a scent that interests them. In some other mammals, the entire organ contracts or pumps in order to draw in the scents [12].

The presence and role of the VNO in humans remains controversial. Some reports state that it completely regresses during fetal development, while others are emphatic regarding its presence in humans [16,17]. Its function is stated to be similar as in animals, i.e., the ability to register the presence of pheromones [18].

### Presentation of the hypothesis

We hypothesize that the VNO might be unilaterally, or more often bilaterally removed or irreversibly damaged, during a Le Fort I osteotomy when maxillary advancement, impaction, or extrusion is performed, in order to achieve a good esthetic and/or functional result during orthognathic surgery. During a Le Fort I osteotomy, the VNO, together with the supplying nerve, is excised in the course of dissecting the nasal mucosa from the hard palate osseous base.

As a result of this surgical intervention, the patient loses the ability to recognize all scents of a pheromone nature, which significantly changes sexual preferences and behaviour, including personal criteria for the choice of a mate.

### Evaluation of the hypothesis

#### Why the VNO exists in adult humans

Although some authors dismiss the existence of a true VNO, there are many observations demonstrating a specific organ in the nasal mucosa which is not of a respiratory or olfactory nature [16,17,19]; thus, there should be no doubt that the VNO exists in humans. The VNO is often described as a blind-ending diverticulum in the septal mucosa opening via a depression, called the VNO pit, into the nasal cavity approximately 2 centimetres from the nostril [17].

On the basis of phylogenetic development, it would be surprising if all chemosensory communication has been lost. The fact that chemical communication does not seem to be a strong determinant of human behaviour is not a very strong argument for dismissing vomeronasal function, as implied by Keverne [20] and Meredith [12]. The truth will likely be somewhere in between, i.e., the VNO is developed in adult humans, but has a significantly reduced function during phylogenetic development due to the more rapid development of other senses.

#### Why the VNO exhibits properties of a sensory organ

The VNO in humans does not have the classical appearance of a peripheral sensory ending, from which a bundle of nerve fibres originates and terminates in the central nervous system. It has been shown that adult human vomeronasal epithelium has a limited number of bipolar cells, positive for neuron-specific enolase, a specific marker for cells of neural origin [17,21]. In addition, a significantly higher density of unmyelinated axons has been observed in the mucosa below and near the human VNO, in comparison with other nasal mucosa [22]. On the other hand, axons observed in the mucosa do not reveal the continuity or synaptic contact with the epithelial cells of vomeronasal nature [23].

The innervation of the VNO in animals is quite complex (for a review, see [13]). In the human fetus, as in other species, the terminalis nerve, i.e., the zero cranial nerve, connects the VNO and the brain, acting as a pathway for migration of luteinizing hormone releasing hormone-producing neurons from the region of the VNO epithelium into the brain [24-26]. In addition, the terminalis nerve clearly persists in human adults [12,27]. Although it has not been shown that the terminalis nerve carries the axons of the VNO in adults, such speculation had often been published [12,28,29].

#### Why the VNO responds to pheromones

There is electrophysiologic evidence of a response of the VNO to urine [18]. Jacob and colleagues [30,31] have reported changes in mood in humans elicited by chemicals extracted from human skin, including androstandione and estratetraenyl compounds. It has been reported that a local electrophysiologic response to the application of small amounts of the same substances, confined directly to the vomeronasal region and termed the electrovomeronasogram response [32,33]. In addition, responses from isolated cells and also a systemic response to such an application have been reported. Importantly, conventional odours did not elicit such response [32].

Several indirect reports of the presence of pheromone-like substances, influencing human behaviour, have been published (for a review, see [12]). One of the most notable examples is a trend towards synchronization of menstrual cycles in women who live together [34]. This function might be phylogenetically-based, i.e., the Lee-Boot effect, showing that group-housed female mice suppress estrus in order to conserve the energy normally put into cycling when there is no possibility of pregnancy [35]. Conversely, in the presence of male stimuli, i.e., pheromones, group-housed females return to estrus cycling, the so-called Whitten effect [36]. We can speculate that signalling pheromones might communicate information that alters an individual's probability of responding without necessarily evoking an immediate observable response in humans.

#### How the VNO influences human behaviour

Although the VNO has more functions, probably the most important and most studied function of the VNO is its influence on sexual behaviour. Experiments on animal models have shown that the destruction or deafferentation of the VNO produces severe sexual behavioural deficits in both males and females (for a review, see [13]). Based on a number of studies, the experiments of Bruce [37] most closely support our hypothesis. In these experiments, fertilised eggs failed to implant if a strange male was exchanged for the mating male in the cage of a female within 4 days of copulation. This effect was later shown to be dependent on the functional VNO [38]. These experiments also indicated the important role of memory in the process of recognition of pheromones by the VNO. Recently, there has been a report showing that male mice deficient in Trpc2, an ion channel specifically expressed in VNO neurons and essential for transduction in the VNO, are impaired in sex-discrimination and male-male aggression, whereas females deficient in Trpc2 show a reduction in female-specific behaviour, including maternal aggression and lactating behaviour. In addition, the same results have been observed after the VNO is removed [39].

#### Why the VNO is lost during a Le Fort I osteotomy

Our hypothesis presumes the VNO is lost during maxillary movement. In the case of maxillary impaction, we perform not only bony structure reduction, but also partial reduction of the inferior cartilagineous part of the nasal septum to prevent unfavorable and non-esthetic nose bending. During such a reduction, the VNO is electrocoagulated to control the bleeding from the nasopalatine artery, or even removed *in toto*. This statement is indirectly supported by the findings of Trotier et al. [17], who found a substantially lower number of VNOs in patients who underwent septal surgery of a different nature in comparison with healthy individuals.

#### Why the loss of the VNO influences behaviour

To our knowledge, no comprehensive scientific study has analyzed the role of VNO loss during orthognathic surgery on the post-operative establishment of new relationships and mating behaviour of patients. However, several indirect reports have supported this statement. Jacobson [11] observed a 65% increase in positive influence on personality and self-confidence in patients after orthognathic surgery. In addition, 24% of patients stated an improvement in the establishment of relationships with the opposite sex [11]. Williams et al. [40] showed that of patients who were not aware they had a problem in their social life, 24% stated that their social life was significantly improved after orthognathic surgery.

Importantly, social anxiety and depression rating indices of patients scheduled for esthetic surgery in the facial region, such as a blepharoplasty, face lift, or otoplasty, were significantly higher then the same indices of patients scheduled for orthognathic surgery. Thus, orthognathic patients present a significantly lower grade of psychological vulnerability [41]. In addition, patients having orthognathic surgery seem to have fewer postoperative complications than patients having other cosmetic procedures [42]. Pogrel and Scott [43] conclude that most orthognathic surgery patients are psychologically normal, so any routine psychological or psychiatric preoperative examination is not necessary. These data indicate the orthognathic patients are primarily not expected to change their personal lives dramatically. However, our experiences with more than 1000 patients who underwent combined orthodonthic and orthognathic treatment in our department indicate that the number of patients who changed their social life in terms of a change in existing mates or even became promiscuous is not as low as might be expected (Foltán, unpublished).

### Consequences of the hypothesis and discussion

Our hypothesis presumes there is an inhibitory role of the VNO in terms of identification of inappropriate individuals for mating. This inappropriate individual excretes pheromones which are recognized as inappropriate by the VNO and such information is transduced into limbic brain structures and evokes an involuntary response in terms of not "liking that person" or "feeling something strange." We believe this is a phylogenetically old function for the exclusion of inappropriate mates for the conceiving of descendants and their additional care. The pre- and post-delivery care of the mother, which is important for females, but also the need for the proper selection of a mother capable of giving birth to healthy children, which is important for males, were and are more important in humans than in, for example, mice, because the time needed for the pregnancy and also the lactation period are quite long.

Kimchi et al. [39] recently showed that the murine VNO-mediated pheromone inputs act in wild-type females to repress male behaviour and activates female behaviour. On the basis of these results, they concluded that functional neuronal circuits underlying male-specific behaviour exist in the normal female mouse brain [39]. Thus, when applied to our hypothesis, when the inhibitory mechanism, i.e., the VNO, is destroyed, the mechanism of negative feedback is disrupted and the individual is free to choose a partner for mating; however, he/she might prefer more than one individual and therefore suddenly becomes promiscuous.

On the basis of our hypothesis, a comprehensive study of orthognathic patients with a focus on social life, together with the number and gender of mates, analyzed before and after surgery, might be developed. Importantly, patients who underwent a Le Fort I osteotomy should be evaluated separately from those who underwent a sagittal split osteotomy of the mandible and/or a genioplasty without maxillary advancement and/or set back.

## Conclusion

Our hypothesis presumes that the loss of the VNO during orthognathic surgery might influence the post-operative social life of patients in terms of a loss of negative feedback, which is important for exclusion of inappropriate mates.

## Competing interests

The authors declare that they have no competing interests.

## Authors' contributions

Both authors made substantial contributions to conception, design, and analysis and interpretation of data, both have been involved in drafting the manuscript or revising it critically for important intellectual content, and have given final approval of the version to be published.
